# A Signature Inferred from *Drosophila* Mitotic Genes Predicts Survival of Breast Cancer Patients

**DOI:** 10.1371/journal.pone.0014737

**Published:** 2011-02-28

**Authors:** Christian Damasco, Antonio Lembo, Maria Patrizia Somma, Maurizio Gatti, Ferdinando Di Cunto, Paolo Provero

**Affiliations:** 1 Molecular Biotechnology Center and Department of Genetics, Biology and Biochemistry, University of Turin, Turin, Italy; 2 Dipartimento di Biologia e Biotecnologie, and Istituto di Biologia e Patologia Molecolari del CNR, “Sapienza” Università di Roma, Roma, Italy; University Medical Center Maastricht, Netherlands

## Abstract

**Introduction:**

The classification of breast cancer patients into risk groups provides a powerful tool for the identification of patients who will benefit from aggressive systemic therapy. The analysis of microarray data has generated several gene expression signatures that improve diagnosis and allow risk assessment. There is also evidence that cell proliferation-related genes have a high predictive power within these signatures.

**Methods:**

We thus constructed a gene expression signature (the DM signature) using the human orthologues of 108 *Drosophila melanogaster* genes required for either the maintenance of chromosome integrity (36 genes) or mitotic division (72 genes).

**Results:**

The DM signature has minimal overlap with the extant signatures and is highly predictive of survival in 5 large breast cancer datasets. In addition, we show that the DM signature outperforms many widely used breast cancer signatures in predictive power, and performs comparably to other proliferation-based signatures. For most genes of the DM signature, an increased expression is negatively correlated with patient survival. The genes that provide the highest contribution to the predictive power of the DM signature are those involved in cytokinesis.

**Conclusion:**

This finding highlights cytokinesis as an important marker in breast cancer prognosis and as a possible target for antimitotic therapies.

## Introduction

A reliable prediction of the outcome of a breast cancer is extremely valuable information for deciding a therapeutic strategy. The analysis of gene expression profiles obtained with microarrays has allowed identification of gene sets, or genetic “signatures”, that are strongly predictive of poor prognosis (see [1], [2] for a recent survey). In the past few years, two types of cancer signatures have been developed, commonly designated as “bottom-up” or “top-down”. In top-down (or supervised) signatures, the risk-predicting genes are selected by correlating the tumor's gene expression profiles with the patients' clinical outcome. One of the most powerful top-down signatures is the so-called 70-gene signature, which includes genes regulating cell cycle, invasion, metastasis and angiogenesis [3]. This signature outperforms standard clinical and histological criteria in predicting the likelihood of distant metastases within five years [4]. Although highly predictive of cancer outcome, top-down signatures have the drawback of including different gene types, thereby preventing precise definition of the biological processes altered in the tumor.

Bottom-up (or unsupervised) signatures are developed using sets of genes thought to be involved in specific cancer-related processes and do not rely on patients' gene expression data. Examples of these signatures are the “Wound signature” that includes genes expressed in fibroblasts after serum addition with a pattern reminiscent of the wound healing process [5], [6], the “Hypoxia signatures” that contains genes involved in the transcriptional response to hypoxia [7]-[9], and the “Proliferation signatures” that include genes expressed in actively proliferating cells [10], [11]. Other bottom-up signatures are the “Embryonic Stem cells (ES) signature” [12], the proliferation, immune response and RNA splicing modules signature [13] (henceforth abbreviated as “Module signature”) the “invasiveness gene signature” (IGS) [14] and the chromosomal instability signature (CIN) [15]. The “ES signature” is based on the assumption that cells with tumor-initiating capability derive from normal stem cells. This signature reflects the gene expression pattern of embryonic stem cells (ES) and includes genes that are preferentially expressed or repressed in this type of cells [12]. The “Module signature” was generated by selecting gene sets that were enriched in nine pre-existing signatures, and consists of gene modules involved in 11 different processes including the immune response, cell proliferation, RNA splicing, focal adhesion, and apoptosis [13]. The IGS signature includes genes that are differentially expressed in tumorigenic breast cancer cells compared to normal breast-epithelium cells; the 186 genes of this signature are involved in a large variety of cellular functions and processes [14]. The CIN signature has features of both top-down and bottom-up signatures; it was developed by selecting genes with variations in the expression level correlated with the overall chromosomal aneuploidy of tumor samples [15].

Tumors are characterized by frequent mitotic divisions and chromosome instability. In addition, several independent studies have shown that mitotic activity in breast cancer samples from lymph node-negative patients positively correlates with poor prognosis [16]-[19]. We thus reasoned that genes required for mitotic cell division and genes involved in the maintenance of chromosome integrity could be used to develop a new cancer signature. In a recent RNAi-based screen performed in *Drosophila* S2 cells [20], we identified 44 genes required to prevent spontaneous chromosome breakage and 98 genes that control mitotic division. Thus, considering the strong phylogenetic conservation of the mitotic process, rather than relying on functional annotation databases, we used the 142 *Drosophila* genes identified in the screen [20] to develop a new bottom-up signature that includes genes involved in cell division but not yet annotated in the literature. 108 of these 142 *Drosophila* genes have unambiguous human orthologs. Here we show that these 108 human genes constitute an excellent signature to predict breast cancer outcome. This *Drosophila mitotic signature*, or “DM signature”, has minimal overlap with pre-existing gene signatures and outperforms most of them in predictive power.

## Materials and Methods

### Definition of the DM signature

The 142 *D. melanogaster* mitotic genes described in [20] were first converted into Entrez gene ids (file gene_info.gz downloaded from the Entrez Gene ftp site in June 2008). We then used Homologene, build 62, to obtain the 108 human orthologues that compose the DM signature. We considered only one-to-one orthology relationships reported in Homologene. This criterion led to the exclusion from the DM signature of several human genes that are commonly considered homologous to the *Drosophila* genes. However, the degree of homology between these human genes and their *Drosophila* counterparts was not sufficient for inclusion in Homologene.

### Breast cancer datasets

We used the following publicly available breast cancer datasets: NKI [4]; Pawitan ([21] - Gene Expression Omnibus (GEO-) series GSE1456); Miller ([22] - GEO series GSE3494); Sotiriou ([23] - GEO series GSE2990); Desmedt ([24] - GEO series GSE7390); and Wang ([25] - GEO series GSE2034). We used relapse-free survival times when available, and overall survival times otherwise. Since the Sotiriou, Desmedt and Miller datasets have some patients in common, we merged the Sotiriou and Desmedt datasets in a single dataset, from which we removed the patients included in the Miller dataset. We refer to this combined dataset as the Sotiriou-Desmedt dataset. Normalized expression data and clinical data for the NKI dataset were obtained from http://www.rii.com/publications/2002/nejm.html. For the Affymetrix-based datasets, we obtained gene expression values from the raw data, using MAS 5.0 algorithm as implemented in the Simpleaffy [26] package of Bioconductor [27]. For all datasets we considered only the probesets unambiguously assigned to one Entrez Gene ID in the platform annotation. For the Affymetrix platform, we used the annotation provided by the manufacturer, version 25, which allowed us to identify single or multiple probesets for 105 of the 108 DM signature genes. For the NKI dataset we used the annotation file provided in the website mentioned above; the correspondence between sequence accession number and Entrez gene was obtained from the Entrez gene ftp site; 98 of the 108 DM genes were thus associated with one or multiple probes.

### Determination of the predictive power of the genes in the DM signatures by clustering analysis

To determine whether the expression profiles of the genes included in the DM signature are significantly and robustly correlated with the disease outcome we used the following procedure on the datasets mentioned above: (a) selecting the microarray probes unambiguously associated to the signature genes; (b) creating two groups of patients by Pearson correlation-based hierarchical clustering, using only the expression profiles of the probes selected in step a; (c) determining by a standard log-rank test, as implemented in the *survival* library of R, whether the cumulative probability of survival is significantly different between the two groups.

### Determination of prognostic scores

For all datasets we divided the patients into two groups (good- and poor-outcome) based on their status at five years. We then calculated the prognostic scores for outcome prediction at five years using the following procedures. For the 70-gene signature, the score of a patient is the cosine-correlation of the expression profile of genes with good-prognosis found in http://www.rii.com/publications/2002/nejm.html [4]. The genes in the signature, given at as accession numbers, were translated into Entrez gene IDs and then into Affymetrix probesets using Affymetrix annotation files, version 25. We obtained 76 probesets for the HG-U133A platform, and 109 probesets for the HG-U133A and HG-U133B platforms considered together. Probesets corresponding to the same gene were assigned the same coefficient in the good-prognosis profile.

For the Wound and IGS signatures, the score of a patient is given by the Pearson correlation of the expression profile of the signature genes. For the Wound signature the core serum response centroid is available at http://microarray-pubs.stanford.edu/wound [5]. The genes in the signature were translated into Entrez gene ids and then into Affymetrix probesets using the procedure described above. We obtained 493 probesets for the HG-U133A platform, and 667 probesets for the HG-U133A and HG-U133B platforms considered together. Probesets corresponding to the same gene were assigned the same expression value in the core serum response centroid. The centroid for the IGS signature is directly given in Affymetrix probesets [14].

For the CIN [15], Proliferation [11] and Hypoxia [9] signatures, the score of a patient is the sum of the logarithmic expression of the signature genes in the patient sample. For the CIN and Proliferation signatures, the gene symbols, were translated first into Entrez gene ids and then into Affymetrix probesets as described above. The Hypoxia signature is directly given in terms of Affymetrix probesets.

For the DM signature, the prognostic score of a patient is given by
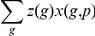
where the sum is over all the probesets associated to the signature, *z(g)* is the *z-*score of probeset *g* computed in the Pawitan dataset and *x(g,p)* is the logarithmic expression level of probeset *g* in patient *p*. The Affymetrix probesets that comprise the DM signature together with their *z-*scores are reported in Table S1.

We used Receiver Operating Characteristic ROC curves to compare the scalable scores on three datasets (Miller, Wang and Sotiriou-Desmedet). The area under the curves and the related standard error were computed using the Hmisc library and programs available at http://biostat.mc.vanderbilt.edu/s/Hmisc. The Pawitan and NKI datasets were not used in this comparison because they were involved in the training of the DM and 70-gene signatures, respectively.

### Contribution of specific gene classes to the predictive power of the signature

The contribution of each probeset *g* to the difference in score between poor- and good-prognosis patients is defined as
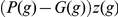
where *P(g)* (*G(g)*) is the logarithmic expression of the probeset averaged on all poor (good) prognosis patients and *z(g)* is the *z-*score of the probeset. Given a subset of the DM signature (*e.g.* cytokinesis-related genes), we used a Mann-Whitney U test to compare the contribution of the probesets included in the subset to the contribution of all the other probesets.

## Results

### Generation of the DM signature

We have recently carried out an RNAi-based screen to detect *Drosophila* genes required for chromosome integrity and for the fidelity of mitotic division [20]. Since these types of genes tend to be transcriptionally co-expressed, we first used a co-expression-based bioinformatic procedure to select a group of 1,000 genes highly enriched in mitotic functions. We then performed RNAi against each of these genes in *Drosophila* S2 cultured cells. Phenotypic analysis of dsRNA-treated cells allowed the identification of 142 genes representative of the entire spectrum of functions required for proper transmission of genetic information. 44 of these genes were required to prevent spontaneous chromosome breakage. The remaining 98 genes specified a variety of mitotic functions including those required for spindle assembly, chromosome segregation and cytokinesis [20]. Based on the observed RNAi phenotypes, these 142 genes were subdivided into 18 phenoclusters [20].

To construct the DM signature we identified the human homologues of these *Drosophila* genes, according to Homologene [28]. Both the genes required for chromosome integrity and those involved in the mitotic process turned out to be highly conserved in humans. 36 of the 44 chromosome-integrity genes and 72 of the 98 mitotic genes had clear human orthologues. These 108 human genes, and their classification according to the phenotypes associated with RNAi-mediated silencing of their *Drosophila* counterparts, are listed in Tables 1 and S1. Collectively, the genes in Table 1 constitute the DM signature. The remaining 34 *Drosophila* genes identified in the screen [20] were not included in the DM signature because they did not have an unambiguous human homologue in Homologene (Release 62).

**Table 1:** Classification of the 108 genes of the DM signature according to the RNAi phenotypes of their *Drosophila* orthologues. The phenoclusters, indicated in bold characters, are described in detail in [20].

RNAi phenotypes elicited by the *Drosophila* genes	Names of the human orthologues
Chromosome aberrations (**CA**)	*C15orf44*, *CASP7*, *CNOT3*, *CTPS*, *CUL4B*, *CWC15*, *DCAKD*, *DDB1*, *FRG1*, *H3F3A, MSH6*, *ORC5L*, *PCNA*, *PIAS1*, *PPAN-P2RY11*, *POLA1*, *PRIM2*, *PRPF3*, *RAD54L*, *RFC2*, *RPA1*, *RRM2*, *SART1*, *SF3A3*, *SMC1A*, *TAF6*, *TFDP2*, *TK2*, *TPR*, *TYMS*, *WBP11*, *WDR46*, *WDR75*, *XAB2*, *XRN2*, *ZMYM4*.
Abnormal chromosome structure. **CC1,** loss of sister chromatid cohesion in heterochromatin; **CC2** and **CC3**, defective lateral and longitudinal chromosome condensation, respectively	**CC1:** *MCM3, MCM7, SMC3.* **CC2:** *NCAPD2, NCAPG, SMC4, SMC2.* **CC3:** *MASTL, ORC2L, TOP2A.*
Abnormal chromosome segregation. **CS1,** defective chromosome duplication; **CS2**, precocious sister chromatid separation; **CS3** and **CS4**, lack of sister chromatid separation; **CS5,** defective chromosome segregation during anaphase	**CS1:** *CDT1.* **CS2:** *BUB3, KNTC1, ZW10.* **CS3** and **CS4:** *ASCC3L1, CCNB1, CDC40, DHX8, KIAA1310, LSM2, PRPF31, SF3A1, SF3A2, SF3B1, SF3B2, SF3B14, SLU7, SNRPA1, SNRPE, TXNL4A, U2AF1*, *U2AF2.* **CS5:** *ANAPC5, ANAPC10, CDC20, KIF4A, KIN*, *PSMC1, SFRS15.*
Abnormal spindle morphology: **SA1,** short spindles; **SA2,** spindles with a low MT density; **SA3,** poorly focused spindle poles, **SA4** miscellaneous spindle defects	**SA1:** *CKAP5, EIF3A, EIF3D, EIF3E, EIF3I, GTF3C3, MAPRE3, NOC3L, RRP1B, TBK1, THOC2, TUBB2C, WDR82.* **SA2:** *TRRAP, TUBGCP4, TUBG2.* **SA3:** *ASPM, CENPJ, MKI67IP, PPP1R8.* **SA4:** *CDC2, KIFC1, KIF11, KIF18A.*
Abnormal spindle and chromosome structure: **SC1,** defective chromosome condensation and cytokinesis; **SC2,** multiple mitotic defects	**SC1:** *AURKC, RBBP7.* **SC2:** *PLK1.*
Frequent cytokinesis failures: **CY1** and **CY2**, defective in early and late cytokinesis, respectively	**CY1:** *ECT2, KIF23, PRC1, RACGAP1.* **CY2:** *ANLN, CIT.*

The DM signature shares very few genes with pre existing signatures. We considered the top- down 70-gene signature [3] and several bottom-up signatures based on various aspects of cancer biology: the Wound signature [5], [6]; the ES signature [12]; the IGS signature [14] the Hypoxia signatures of Sung et al. [8] and Winter et al. [9]; the Proliferation signature of Starmans et al. [11]; the proliferation/immune response/RNA splicing (Module) signature [13] and the chromosomal instability (CIN) signature [15]. The number of genes that the DM signature shares with the 70-gene, ES, IGS, Wound and Hypoxia signatures is extremely small. The overlap is higher with the Module, Proliferation and CIN signatures, but none of these signatures shares more than 20% of its genes with the DM signature (Table 2).

**Table 2:** The DM signature shares very few genes with other major cancer signatures.

*Signature*	*# of genes in the signature*	*Genes in common with the DM signature*
Module	261	18 (6.9%)
CIN	71	14 (19.7)
ES	1029	14 (1.4%)
Wound	371	6 (1.6%)
Proliferation	52	6 (11.5%)
70-gene	61	2 (3.3%)
Hypoxia (Winter)	92	2 (2.2%)
IGS	175	2 (1,1%)
Hypoxia (Sung)	126	1 (0.8%)

25 of the 108 genes of the DM signature are included in the list of genes periodically expressed during the cell cycle in HeLa cells [10], compared to 5.8 expected by chance (P = 2.2E-10). Thus, as expected for genes involved in cell division, a substantial fraction of the DM signature genes has a cell cycle-dependent expression.

### The prognostic value of the DM signature

For a preliminary assessment of the predictive power and robustness of the DM signature we used six publicly available breast cancer datasets: (i) NKI, which contains expression data from primary breast tumors of 295 consecutive, relatively young (age<52 yrs) patients [4]; (ii) Pawitan, which includes data from 159 consecutive breast cancer patients [21]; (iii) Miller, with data from 251 patients selected from a consecutive series based on the quality of the material [22]; (iv) Desmedt and (v) Wang, which contains expression data from 198 and 286 lymph-node negative, systemically untreated patients, respectively [24], [25]; (vi) Sotiriou, which includes 189 invasive breast carcinomas [23]. Due to the presence of common samples, we merged the Desmedt and Sotiriou datasets into a single one and removed from it the patients that were also included in the Miller dataset. All datasets contain both ER-positive and ER-negative samples.

**Figure 1:**
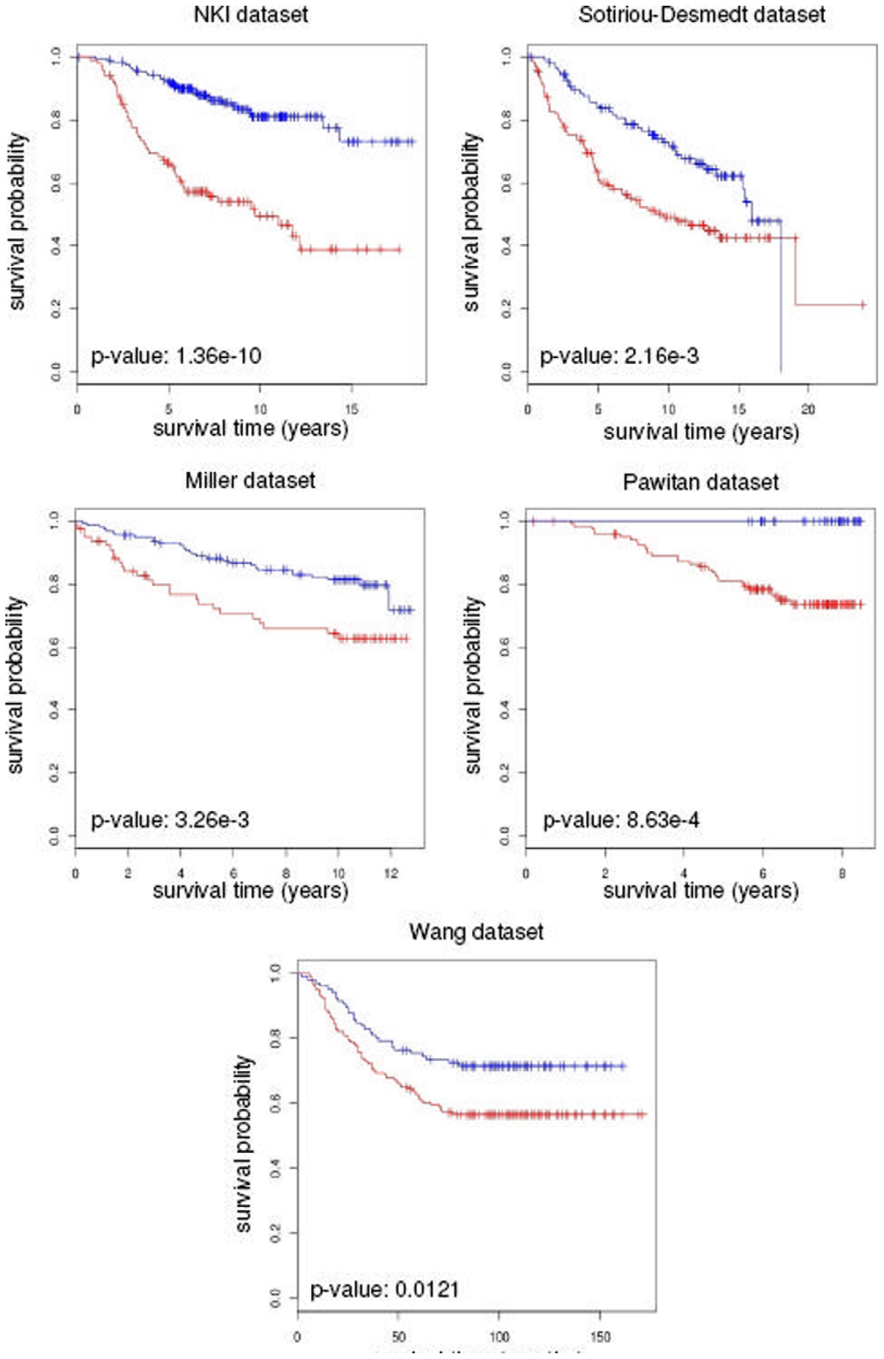
Predictive power of the DM signature. Kaplan-Meier analysis using the DM signature shows significant differences in survival of patients from five independents breast cancer datasets.

Although most of these gene expression data were generated using the same microarray platform, and could in principle be merged in a single dataset as recently described [13], we evaluated the DM signature on the individual datasets. We chose this approach because the robustness of a gene signature on independent datasets is an important criterion for validation of its predictive power. In our prognostic power analysis, we used relapse-free survival times when available, or overall survival times otherwise. Because three genes of the DM signature (*H3F3A, PPAN-P2RY11 and KIF4)* were not represented in the Affymetrix platform, we performed our analyses on 105 genes. For each dataset, patients were divided into two groups based on the expression profiles of the genes in the DM signature using hierarchical clustering. Differences in survival probability between the two groups were then evaluated with a standard log-rank test on Kaplan-Meier curves. Figure 1 shows that the differences in survival are statistically significant for all datasets considered.

As mentioned above, the DM signature contains two broad classes of genes, namely 72 mitotic genes (71 in platform) and 36 genes required for the maintenance of chromosome integrity (34 in platform). To determine the relative contribution of these two gene classes to the predictive power of the DM signature, we performed the analysis using the two categories of genes separately. Both gene groups turned out to be independently predictive of survival (Figure S1). However the predictive power of the global signature was higher in all cases.

We also asked whether the DM signature is predictive of survival in other tumors besides breast cancer. Using the hierarchical clustering approach described above, we found that the DM signature is predictive of survival in a large lung cancer dataset [29] (*P* = 3e−6) and in a glioma dataset [30] (*P* = 0.0170). However, the DM signature is not significantly predictive in other lung cancer [31] and glioma [32] datasets, and in renal [33] or ovarian [31] cancer datasets. The p-values of the log-rank tests for non-breast datasets are reported in Table S2.

### Evaluation of a prognostic score for the DM signature

Subdivision of patients into risk groups using the unsupervised clustering-based approach described above allows assessment of the predictive power of a gene signature, but does not allow specificity (fraction of low-risk patients correctly classified) and sensitivity (fraction of high-risk patients correctly classified) to be tuned according to specific requirements. However, such tuning is important in clinical applications, because the misclassification of a high-risk patient is potentially more harmful than the misclassification of a low-risk patient. Indeed, the 70-gene signature [3], which is used in clinical practice, assigns a risk score to each patient; patients are then classified based on a score threshold that can be tuned to obtain the desired compromise between specificity and sensitivity. Scalable prognostic scores, each computed from gene expression data with a specific algorithm, have been previously defined also for the Wound [6], IGS [14], Proliferation [11], CIN [15] and Hypoxia [9] signatures.

We determined a scalable prognostic score for the DM signature, using a procedure similar to that employed by Wang and co-workers [25]. We define the DM prognostic score as the sum of the logarithmic expression values of the signature genes, each multiplied by its *z-*score. The Cox *z-*score measures the correlation between the expression pattern of a gene and survival of the patient. A positive (negative) *z-*score indicates negative (positive) correlation between the gene expression level and patient's survival time.

We used the Pawitan dataset as training set and computed the Cox *z-*scores for the Affymetrix probesets associated with the DM signature (the *z-*scores of all probesets are shown in Table S1). The distribution of these *z-*scores is consistently shifted towards positive values compared to the distribution of the *z-*scores of all genes represented on the microarrays (P-values between 1.1e-6 and 3.3e-15 from one-sided Mann-Whitney U test) (Figure S2). Thus, as expected for proliferation-related genes, for most genes in the DM signature an increased expression level is negatively correlated with survival.

**Figure 2:**
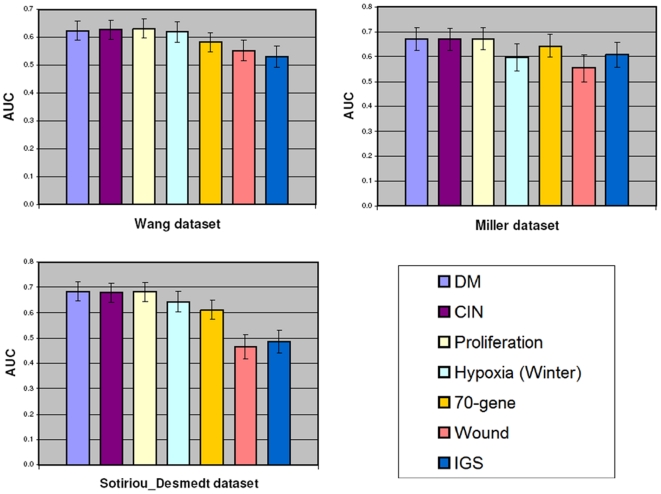
Comparative evaluation of the prognostic score of the DM signature. The prognostic score of the DM signature is compared to those obtained from the CIN [15], Proliferation [11], IGS [14], Hypoxia [9], 70-gene [3], and Wound [5] signatures in the three datasets not used for training. The scores are used to predict outcome at five years. The bars show the areas under the ROC curves (AUC).

We then compared the DM signature score with the scores of 6 other scalable signatures for performance in predicting cancer outcome at 5 years. For this analysis we used ROC curves generated with the Affymetrix datasets not employed for training (Miller, Sotiriou-Desmedt and Wang). The scores of the CIN [15], Proliferation [11], 70-gene [3], Wound [6], IGS [14], and Hypoxia [9] signatures were computed as described in the respective references, after mapping the genes to the Affymetrix platform (see Methods for details). As shown in Figure 2, the predictive power of the 3 proliferation-based signatures (DM, CIN and Proliferation), measured by the Area Under ROC Curves (AUC), is very similar in all datasets and systematically higher than that of the 70-gene, Wound, IGS, or Hypoxia signature.

Since the DM signature and the two other proliferation-based signatures perform similarly in predicting outcome at 5 years (see Fig. 2), we compared their performance in greater detail at three sensitivity values. In Table 3, we show for each signature and dataset both the specificity and the P-value of the log-rank test that compares the survival probabilities of the two groups of patients identified by the signature. These parameters provide different assessments of the predictive power: while the specificity refers to the ability of the signature to predict the outcome only at the 5-year endpoint, the P-value takes into account the complete survival curves, and thus measures the ability to stratify the patients over the whole time range. The results in Table 3 show that the DM signature performs slightly better than the other two signatures at the higher sensitivities, especially in terms of P-value. The differences in performance between the three signatures are driven by the fraction of patients that are discordantly classified in the different signatures. These fractions, which range from ∼2% to ∼10% in the three datasets, are reported in Table S3.

**Table 3:** Comparison of the performances of the proliferation-based signatures.

90% sensitivity	DM		CIN		Proliferation		
	P value	Specificity	P value	Specificity	P value	Specificity	
Miller	**2.26E-04**	0.318	5.44E-04	**0.352**	4.89E-04	**0.352**	
Sotiriou-Desmedt	**4.44E-03**	**0.335**	0.0312	0.329	0.0124	0.329	
Wang	**4.08E-03**	0.226	0.0114	**0.260**	0.015	0.227	
70% sensitivity	DM		CIN		Proliferation		
	P value	Specificity	P value	Specificity	P value	Specificity	
Miller	**1.77E-04**	**0.614**	7.63E-03	0.523	3.02E-03	0.562	
Sotiriou-Desmedt	4.51E-04	**0.613**	**4.25E-04**	0.600	1.24E-03	0.574	
Wang	**4.25E-04**	**0.547**	5.58E-04	**0.547**	1.19E-03	0.536	
50% sensitivity	DM		CIN		Proliferation		
	P value	Specificity	P value	Specificity	P value	Specificity	
Miller	**3.91E-04**	**0.733**	8.81E-04	0.705	1.42E-03	0.716	
Sotiriou-Desmedt	0.138	0.697	**0.134**	**0.722**	0.161	0.690	
Wang	6.85E-03	0.669	**2.41E-03**	**0.691**	0.022	0.641	

The best performing signature in terms of specificity or P-value is shown in bold.

We also performed multivariate Cox analysis to ascertain whether the DM signature predicts survival independently of other molecular and clinical tumor markers. The results for the Miller dataset (Table 4), which is the richest in clinical annotations, and those for the other datasets (Table S4) clearly show that the DM score is a predictor independent of several tumor parameters. Multivariate Cox analysis on the Miller dataset showed that also the other proliferation-based signatures are independent of the same parameters considered for the DM signature (Table S5).

**Table 4:** Multivariate Cox analysis for the Miller dataset shows that the DM score is predictive of survival independently of other molecular and clinical tumor markers.

Covariate	Odd ratio (95% C.I.)	P-value
LN (positive = 1, negative = 0)	2.82 (1.53–5.21)	8.95E-04
DM score (range 0–10)	1.32 (1.08–1.60)	0.0057
Size (mm)	1.04 (1.01–1.06)	0.0065
ER (positive = 1, negative = 0)	3.34 (1.11–10.00)	0.031
Age (years)	1.02 (1.00–1.04)	0.057
PGR (positive = 1, negative = 0)	0.53 (0.23–1.23)	0.14
P53 (mutant = 1, wt = 0)	0.97 (0.49–1.95)	0.95
Grade (1–3)	0.99 (0.56–1.75)	0.96

LN = lymph node status; ER = estrogen receptor status; PGR = progesteron receptor status.

The patients that would benefit the most from an effective prognostic predictor are those with lymph-node negative breast cancers. The Wang dataset includes only lymph-node negative patients, while the Miller and Sotiriou-Desmedt datasets include both node-positive and negative patients. Therefore we evaluated the performance of the DM signature on the patients of the latter datasets by computing the AUC under ROC curves at the five-year endpoint. For both the Miller and the Sotiriou-Desmedt studies, the AUC values obtained for the lymph node-negative patients were very similar to the values obtained for the entire datasets (0.616 *vs* 0.67, and 0.678 *vs* 0.683, respectively). Thus, we conclude that the DM signature is a robust predictor of survival in lymph-node negative patients.

### Contribution of specific genes and gene classes to the predictive power of the DM signature

We next asked whether any of the phenotypic classes identified by the RNAi screen (chromosome condensation, chromosome integrity, chromosome segregation, spindle assembly and cytokinesis) [20] is especially relevant in separating poor- from good-prognosis patients. We computed the contribution of each probeset in the DM signature to the difference in score between poor- and good-outcome patients (see Methods); we then compared the contribution of specific gene classes to the total score of the 105 genes of the DM signature. For the three Affymetrix datasets not used as training, the cytokinesis genes (*ANLN, CIT, ECT2, KIF23, PRC1, RACGAP1*) turned out to contribute to the difference in score significantly more than other genes (P-values between 0.0025 and 0.012, two-sided Mann-Whitney U test). The function of these genes is highly conserved, as they are required for cytokinesis in both *Drosophila* and humans (reviewed in [34]). Interestingly, high *z-*scores were also observed for *ASPM, KIF18A* and *PLK1* (Table S1). The *Drosophila* homologues of these genes (*asp, Klp67 and polo*) are involved in multiple mitotic stages and are required for cytokinesis [34]. In addition there is evidence that *ASPM* and *PLK1* are involved in human cell cytokinesis [34]. Thus, it appears that cytokinesis genes have higher prognostic value than other mitotic genes and genes required for chromosome integrity.

In the DM signature, there are a few genes whose expression is positively correlated with survival (Table S1). The gene with the most negative *z-*score is *PIAS1* (*z* = −4.07, averaged on two probesets), an E3 ligase involved in sumoylation of DNA repair proteins including BRCA1 [35]. Remarkably, it has been recently shown that the expression of this gene is substantially reduced in colon cancers [36].

## Discussion

We have shown that the DM signature is highly predictive of survival in five major breast cancer datasets. The DM signature contains two classes of genes required for cell proliferation: genes that maintain the integrity of mitotic chromosomes and genes that mediate mitotic division. Cell proliferation-associated genes have been previously used to construct several cancer signatures, and large subsets of this type of genes are included in most supervised signatures [37]. Thus, it has been suggested that genes required for cell proliferation may underlie the prognostic power of many cancer signatures [37].

Consistent with this idea, we found that the DM signature has a predictive power for breast cancer outcome similar to that of two other proliferation-based signatures, the CIN signature [15] and the Proliferation signature of Starmans et al. [11]. In addition, we showed that the DM signature outperforms 4 additional signatures that contain different proportions of proliferation-related genes, the Hypoxia [9] the Wound [5], [6], the IGS [14] and 70-gene signature, which is currently used in clinical practice [3]. Altogether, these results indicate that the signatures enriched in proliferation genes are the most powerful predictors of breast cancer outcome.

What is the basis of the high prognostic value of the DM signature and why does it outperform many of the extant signatures? We propose that the high performance of the DM signature reflects its specifically high content in genes truly involved in cell proliferation. The proliferation-associated genes in other signatures have been selected on the basis of their periodic expression pattern during the cell cycle and include several genes that, although periodically expressed, are not involved in basic cell cycle processes [10], [37]. In contrast, genes predicted to play a conserved role in either the maintenance of chromosome integrity or mitosis, are expected to be essential for cell cycle progression and cell proliferation. The expression of these genes should therefore reflect the cell proliferation rate within a cancer better than the gene sets of the other signatures. Consistent with this idea, we have shown that most of the DM signature genes with a high predictive power display increased expression in poor outcome patients (Figure S2).

The idea that survival of breast cancer patients is negatively correlated with the frequency of dividing cells within a tumor sample is not novel. Indeed, it has been shown that a correct measure of the mitotic activity [16], [19] can accurately identify high-risk cases among lymph node-negative patients. However, to be effective, the analysis of mitotic activity must be carried out by well-trained personnel, using a strictly defined protocol [16], [19]. On the other hand, measuring gene expression in tumor biopsies, might not take into account intra-tumor heterogeneity [16], although it might be technically less demanding. We do not know how prognostic values obtained by cytological analysis of mitotic activity compare with values obtained with the DM signature or with the other proliferation signatures. Unfortunately, in the available studies where both mitotic activity and gene expression have been determined in the same tumor sample [4], [11], the mitotic activity was not measured by protocols of proved reliability [38], preventing a direct comparison. We believe that future studies addressing this point will be instrumental to refine our tools for risk assessment in lymph node-negative patients.

We have shown that a group of genes required for cytokinesis (*ANLN, CIT, ECT2, KIF23, PRC1*, *RACGAP1, ASPM, KIF18A* and *PLK1*) contributes to the predictive power of the DM signature significantly more than the other genes. All cytokinesis genes display high positive *z-*scores, indicating that their increased expression is negatively correlated with survival. Strikingly, there is evidence that *ANLN*, *ECT2*, *PRC1*, *RACGAP1*, *ASPM*, and *PLK1* are upregulated in a variety of human cancers and that their overexpression often correlates with poor outcome (see for example [39]-[47] and references therein). In addition, it has been shown that two of these genes, *ETC2* and *ANLN*, are amplified in cancer cells [42], [48]. These findings raise the question of why cytokinesis genes have a higher prognostic value and tend to be more upregulated poor prognosis patients compared to other mitotic genes. It is possible that overexpression of cytokinesis genes is an oncogenic factor per se. However, the finding that *PRC1* overexpression does not result in cell growth enhancement [45] argues against this possibility. Another possibility is that cytokinesis proteins are limited in amount or stability compared to other mitotic proteins. That is, when cell proliferation is strongly enhanced, normal levels of gene transcription and translation would not be sufficient to produce the amounts of cytokinesis proteins required for proper execution of the process. As a result, cancers cell clones overexpressing cytokinesis genes would be favoured over clones in which these genes are normally expressed. This hypothesis is very attractive but it is not sufficiently supported by current data. Further experiments will be required to examine the role of cytokinesis genes in cancer development. For example, one could produce stably transformed cancer-derived cells and ask whether overexpression of cytokinesis genes confers growth advantage compared to overexpression of other types of mitotic genes.

Our study indicates that the DM signature improves risk stratification for breast cancer patients compared to the major extant signatures. In addition, the identification of new cancer prognostic genes with well-defined biological functions, such as those of the DM signature, provides valuable information for development of new prognostic tools based on gene expression. For example, according to a previous approach [6], [11], [13] the genes of the DM signature could be merged with those of other signatures to further improve risk stratification. Finally, our finding that cytokinesis genes tend to be overexpressed in patients with poor prognosis sets forth this class of genes and their protein products as targets for antimitotic therapies.

## Supporting Information

Figure S1 Predictive power of the mitotic and chromosome-integrity genes of the DM signature. Kaplan-Meier survival analysis was performed on five breast cancer datasets using either the 34 chromosome integrity genes or the 71 mitotic genes of the DM signature represented in the Affymetrix platform.
(0.07 MB PDF)

Figure S2 Distribution of the z-scores of the genes of the DM signature compared to the distribution of z-scores of all genes represented in five breast cancer datasets. Distribution of the z-scores of the genes of the DM signature compared to the distribution of z-scores of all genes represented in five breast cancer datasets. The z-scores were obtained using Cox univariate analysis. Note that the distribution of the signature genes is shifted towards positive values.
(0.28 MB PDF)

Table S1 Ranking of the Affymetrix probesets of the DM signature according to their z-scores. The Affymetrix probesets associated with the DM signature genes are ranked according to their Cox z-score computed on the training dataset (Pawitan). The contribution to the difference in score between poor and good prognosis patients in the other datesets is also reported. The phenoclusters associated with the Drosophila genes [20] are abbreviated as follows: CA, chromosome aberrations; CC1, loss of sister chromatid cohesion in heterochromatin; CC2 aberrant lateral chromosome condensation; CC3, aberrant longitudinal chromosome condensation; CS1, defective chromosome duplication; CS2, precocious sister chromatid separation; CS3 and CS4, lack of sister chromatid separation; CS5, defective chromosome segregation during anaphase; SA1, short spindles; SA2, spindles with a low MT density; SA3, poorly focused spindle poles; SA4 miscellaneous spindle defects; SC1, defective chromosome condensation and cytokinesis; SC2, multiple mitotic defects; SC1, defective in early cytokinesis; SC2, defective in late cytokinesis.
(0.06 MB XLS)

Table S2 Predictive power of the DM signature in cancers other than breast. The P-values were obtained from the log-rank test by comparing the cumulative probability of survival of clusters of patients in other cancer types.
(0.01 MB XLS)

Table S3 Differently classified patients by the three proliferation-based signatures. For each dataset and pair of proliferation-based signatures, we report the number of patients classified in different outcome groups, using score cutoffs corresponding to the same sensitivity.
(0.01 MB XLS)

Table S4 Cox multivariate analysis for the NKI, Sotiriou-Desmedt and Wang datasets. The analysis shows that the DM signature is a predictor independent of several clinical parameters.
(0.01 MB XLS)

Table S5 Cox multivariate analysis for the Miller dataset. The analysis shows that the CIN and Proliferation signatures are predictors independent of several clinical and molecular parameters.
(0.01 MB XLS)

## References

[1] Dupuy A, Simon RM (2007). Critical review of published microarray studies for cancer
outcome and guidelines on statistical analysis and
reporting.. J Natl Cancer Inst.

[2] Wirapati P, Sotiriou C, Kunkel S, Farmer P, Pradervand S (2008). Meta-analysis of gene expression profiles in breast cancer:
toward a unified understanding of breast cancer subtyping and prognosis
signatures.. Breast Cancer Res.

[3] van't Veer LJ, Dai H, van de Vijver MJ, He YD, Hart AA (2002). Gene expression profiling predicts clinical outcome of breast
cancer.. Nature.

[4] van de Vijver MJ, He YD, van't Veer LJ, Dai H, Hart AA (2002). A gene-expression signature as a predictor of survival in breast
cancer.. N Engl J Med.

[5] Chang HY, Sneddon JB, Alizadeh AA, Sood R, West RB (2004). Gene expression signature of fibroblast serum response predicts
human cancer progression: similarities between tumors and
wounds.. PLoS Biol.

[6] Chang HY, Nuyten DS, Sneddon JB, Hastie T, Tibshirani R (2005). Robustness, scalability, and integration of a wound-response gene
expression signature in predicting breast cancer survival.. Proc Natl Acad Sci U S A.

[7] Chi JT, Wang Z, Nuyten DS, Rodriguez EH, Schaner ME (2006). Gene expression programs in response to hypoxia: cell type
specificity and prognostic significance in human cancers.. PLoS Med.

[8] Sung FL, Hui EP, Tao Q, Li H, Tsui NB (2007). Genome-wide expression analysis using microarray identified
complex signaling pathways modulated by hypoxia in nasopharyngeal
carcinoma.. Cancer Lett.

[9] Winter SC, Buffa FM, Silva P, Miller C, Valentine HR (2007). Relation of a hypoxia metagene derived from head and neck cancer
to prognosis of multiple cancers.. Cancer Res.

[10] Whitfield ML, Sherlock G, Saldanha AJ, Murray JI, Ball CA (2002). Identification of genes periodically expressed in the human cell
cycle and their expression in tumors.. Mol Biol Cell.

[11] Starmans MH, Krishnapuram B, Steck H, Horlings H, Nuyten DS (2008). Robust prognostic value of a knowledge-based proliferation
signature across large patient microarray studies spanning different cancer
types.. Br J Cancer.

[12] Ben-Porath I, Thomson MW, Carey VJ, Ge R, Bell GW (2008). An embryonic stem cell-like gene expression signature in poorly
differentiated aggressive human tumors.. Nat Genet.

[13] Reyal F, van Vliet MH, Armstrong NJ, Horlings HM, de Visser KE (2008). A comprehensive analysis of prognostic signatures reveals the
high predictive capacity of the proliferation, immune response and RNA
splicing modules in breast cancer.. Breast Cancer Res.

[14] Liu R, Wang X, Chen GY, Dalerba P, Gurney A (2007). The prognostic role of a gene signature from tumorigenic
breast-cancer cells.. N Engl J Med.

[15] Carter SL, Eklund AC, Kohane IS, Harris LN, Szallasi Z (2006). A signature of chromosomal instability inferred from gene
expression profiles predicts clinical outcome in multiple human
cancers.. Nat Genet.

[16] Baak JP, Gudlaugsson E, Skaland I, Guo LH, Klos J (2009). Proliferation is the strongest prognosticator in node-negative
breast cancer: significance, error sources, alternatives and comparison with
molecular prognostic markers.. Breast Cancer Res Treat.

[17] Baak JP, van Diest PJ, Janssen EA, Gudlaugsson E, Voorhorst FJ (2008). Proliferation accurately identifies the high-risk patients among
small, low-grade, lymph node-negative invasive breast
cancers.. Ann Oncol.

[18] Skaland I, Janssen EA, Gudlaugsson E, Klos J, Kjellevold KH (2009). Validating the prognostic value of proliferation measured by
Phosphohistone H3 (PPH3) in invasive lymph node-negative breast cancer
patients less than 71 years of age.. Breast Cancer Res Treat.

[19] van Diest PJ, van der Wall E, Baak JP (2004). Prognostic value of proliferation in invasive breast cancer: a
review.. J Clin Pathol.

[20] Somma MP, Ceprani F, Bucciarelli E, Naim V, De Arcangelis V (2008). Identification of Drosophila mitotic genes by combining
co-expression analysis and RNA interference.. PLoS Genet.

[21] Pawitan Y, Bjohle J, Amler L, Borg AL, Egyhazi S (2005). Gene expression profiling spares early breast cancer patients
from adjuvant therapy: derived and validated in two population-based
cohorts.. Breast Cancer Res.

[22] Miller LD, Smeds J, George J, Vega VB, Vergara L (2005). An expression signature for p53 status in human breast cancer
predicts mutation status, transcriptional effects, and patient
survival.. Proc Natl Acad Sci U S A.

[23] Sotiriou C, Wirapati P, Loi S, Harris A, Fox S (2006). Gene expression profiling in breast cancer: understanding the
molecular basis of histologic grade to improve prognosis.. J Natl Cancer Inst.

[24] Desmedt C, Piette F, Loi S, Wang Y, Lallemand F (2007). Strong time dependence of the 76-gene prognostic signature for
node-negative breast cancer patients in the TRANSBIG multicenter independent
validation series.. Clin Cancer Res.

[25] Wang Y, Klijn JG, Zhang Y, Sieuwerts AM, Look MP (2005). Gene-expression profiles to predict distant metastasis of
lymph-node-negative primary breast cancer.. Lancet.

[26] Wilson CL, Miller CJ (2005). Simpleaffy: a BioConductor package for Affymetrix Quality Control
and data analysis.. Bioinformatics.

[27] Gentleman RC, Carey VJ, Bates DM, Bolstad B, Dettling M (2004). Bioconductor: open software development for computational biology
and bioinformatics.. Genome Biol.

[28] Sayers EW, Barrett T, Benson DA, Bolton E, Bryant SH (2010). Database resources of the National Center for Biotechnology
Information.. Nucleic Acids Res.

[29] Shedden K, Taylor JM, Enkemann SA, Tsao MS, Yeatman TJ (2008). Gene expression-based survival prediction in lung adenocarcinoma:
a multi-site, blinded validation study.. Nat Med.

[30] Phillips HS, Kharbanda S, Chen R, Forrest WF, Soriano RH (2006). Molecular subclasses of high-grade glioma predict prognosis,
delineate a pattern of disease progression, and resemble stages in
neurogenesis.. Cancer Cell.

[31] Bild AH, Yao G, Chang JT, Wang Q, Potti A (2006). Oncogenic pathway signatures in human cancers as a guide to
targeted therapies.. Nature.

[32] Freije WA, Castro-Vargas FE, Fang Z, Horvath S, Cloughesy T (2004). Gene expression profiling of gliomas strongly predicts
survival.. Cancer Res.

[33] Zhao H, Ljungberg B, Grankvist K, Rasmuson T, Tibshirani R (2006). Gene expression profiling predicts survival in conventional renal
cell carcinoma.. PLoS Med.

[34] Eggert US, Mitchison TJ, Field CM (2006). Animal cytokinesis: from parts list to
mechanisms.. Annu Rev Biochem.

[35] Galanty Y, Belotserkovskaya R, Coates J, Polo S, Miller KM (2009). Mammalian SUMO E3-ligases PIAS1 and PIAS4 promote responses to
DNA double-strand breaks.. Nature.

[36] Coppola D, Parikh V, Boulware D, Blanck G (2009). Substantially reduced expression of PIAS1 is associated with
colon cancer development.. J Cancer Res Clin Oncol.

[37] Whitfield ML, George LK, Grant GD, Perou CM (2006). Common markers of proliferation.. Nat Rev Cancer.

[38] van Diest PJ, Baak JP, Matze-Cok P, Wisse-Brekelmans EC, van Galen CM (1992). Reproducibility of mitosis counting in 2,469 breast cancer
specimens: results from the Multicenter Morphometric Mammary Carcinoma
Project.. Hum Pathol.

[39] Suzuki C, Daigo Y, Ishikawa N, Kato T, Hayama S (2005). ANLN plays a critical role in human lung carcinogenesis through
the activation of RHOA and by involvement in the phosphoinositide
3-kinase/AKT pathway.. Cancer Res.

[40] Tamura K, Furihata M, Tsunoda T, Ashida S, Takata R (2007). Molecular features of hormone-refractory prostate cancer cells by
genome-wide gene expression profiles.. Cancer Res.

[41] Skrzypski M, Jassem E, Taron M, Sanchez JJ, Mendez P (2008). Three-gene expression signature predicts survival in early-stage
squamous cell carcinoma of the lung.. Clin Cancer Res.

[42] Fields AP, Justilien V (2009). The guanine nucleotide exchange factor (GEF) Ect2 is an oncogene
in human cancer..

[43] Horvath S, Zhang B, Carlson M, Lu KV, Zhu S (2006). Analysis of oncogenic signaling networks in glioblastoma
identifies ASPM as a molecular target.. Proc Natl Acad Sci U S A.

[44] Lin SY, Pan HW, Liu SH, Jeng YM, Hu FC (2008). ASPM is a novel marker for vascular invasion, early recurrence,
and poor prognosis of hepatocellular carcinoma.. Clin Cancer Res.

[45] Shimo A, Nishidate T, Ohta T, Fukuda M, Nakamura Y (2007). Elevated expression of protein regulator of cytokinesis 1,
involved in the growth of breast cancer cells.. Cancer Sci.

[46] Pellegrino R, Calvisi DF, Ladu S, Ehemann V, Staniscia T (2009). Oncogenic and tumor suppressive roles of polo-like kinases in
human hepatocellular carcinoma..

[47] Schmit TL, Zhong W, Setaluri V, Spiegelman VS, Ahmad N (2009). Targeted depletion of Polo-like kinase (Plk) 1 through lentiviral
shRNA or a small-molecule inhibitor causes mitotic catastrophe and induction
of apoptosis in human melanoma cells.. J Invest Dermatol.

[48] Shimizu S, Seki N, Sugimoto T, Horiguchi S, Tanzawa H (2007). Identification of molecular targets in head and neck squamous
cell carcinomas based on genome-wide gene expression
profiling.. Oncol Rep.

