# Supplementary material for: A Signature Inferred from Drosophila Mitotic Genes Predicts Survival of Breast Cancer Patients
Source: PLoS One. 2011 Feb 28;6(2):e14737. doi: 10.1371/journal.pone.0014737 (PMC3046113; doi:10.1371/journal.pone.0014737)
Supplement: Figure S1 — Predictive power of the mitotic and chromosome-integrity genes of the DM signature. Kaplan-Meier survival analysis was performed on five breast cancer datasets using either the 34 chromosome integrity genes or the 71 mitotic genes of the DM signature represented in the Affymetrix platform. (0.07 MB PDF) [file pone.0014737.s001.pdf]

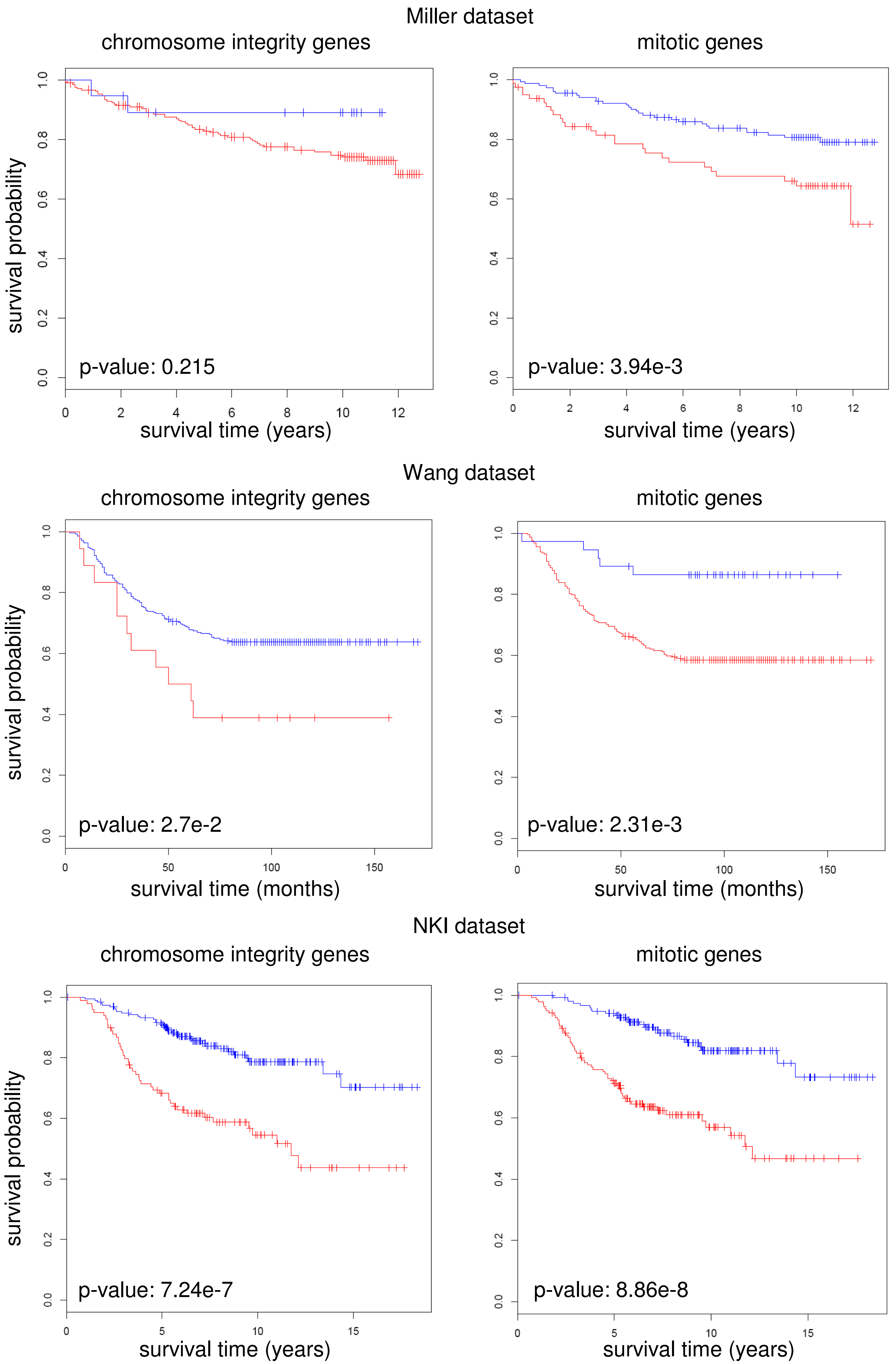
Miller dataset
chromosome integrity genes
mitotic genes
p-value: 0.215
p-value: 3.94e-3
survival probability
survival time (years)
Wang dataset
chromosome integrity genes
mitotic genes
p-value: 2.7e-2
p-value: 2.31e-3
survival probability
survival time (months)
NKI dataset
chromosome integrity genes
mitotic genes
p-value: 7.24e-7
p-value: 8.86e-8
survival probability
survival time (years)

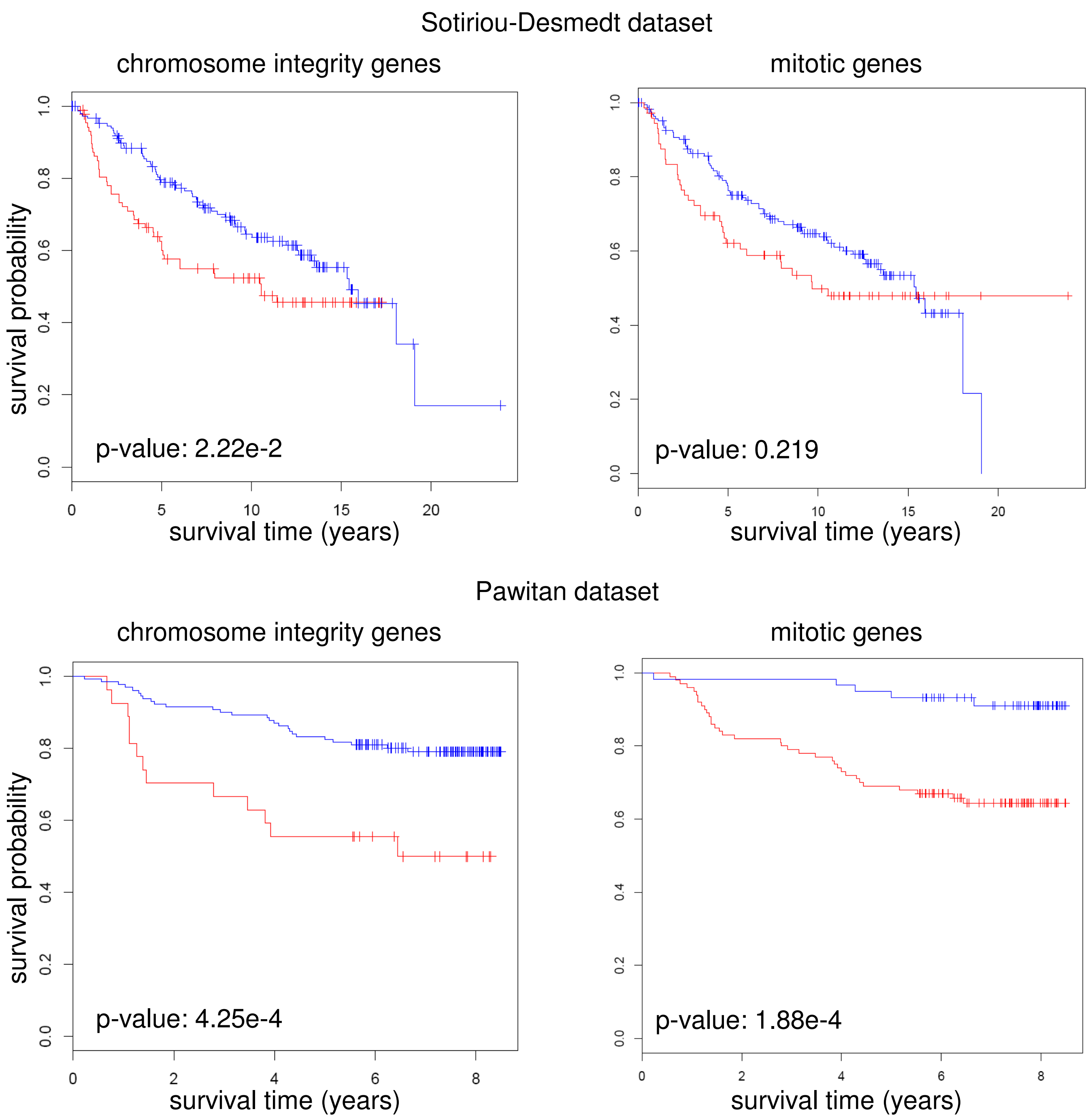

Sotiriou-Desmedt dataset
chromosome integrity genes
mitotic genes
survival probability
p-value: 2.22e-2
p-value: 0.219
survival time (years)
Pawitan dataset
chromosome integrity genes
mitotic genes
p-value: 4.25e-4
p-value: 1.88e-4

Figure S1
