# Supplementary figures and images for: A Signature Inferred from Drosophila Mitotic Genes Predicts Survival of Breast Cancer Patients
Source: PLoS One. 2011 Feb 28;6(2):e14737. doi: 10.1371/journal.pone.0014737 (PMC3046113; doi:10.1371/journal.pone.0014737)

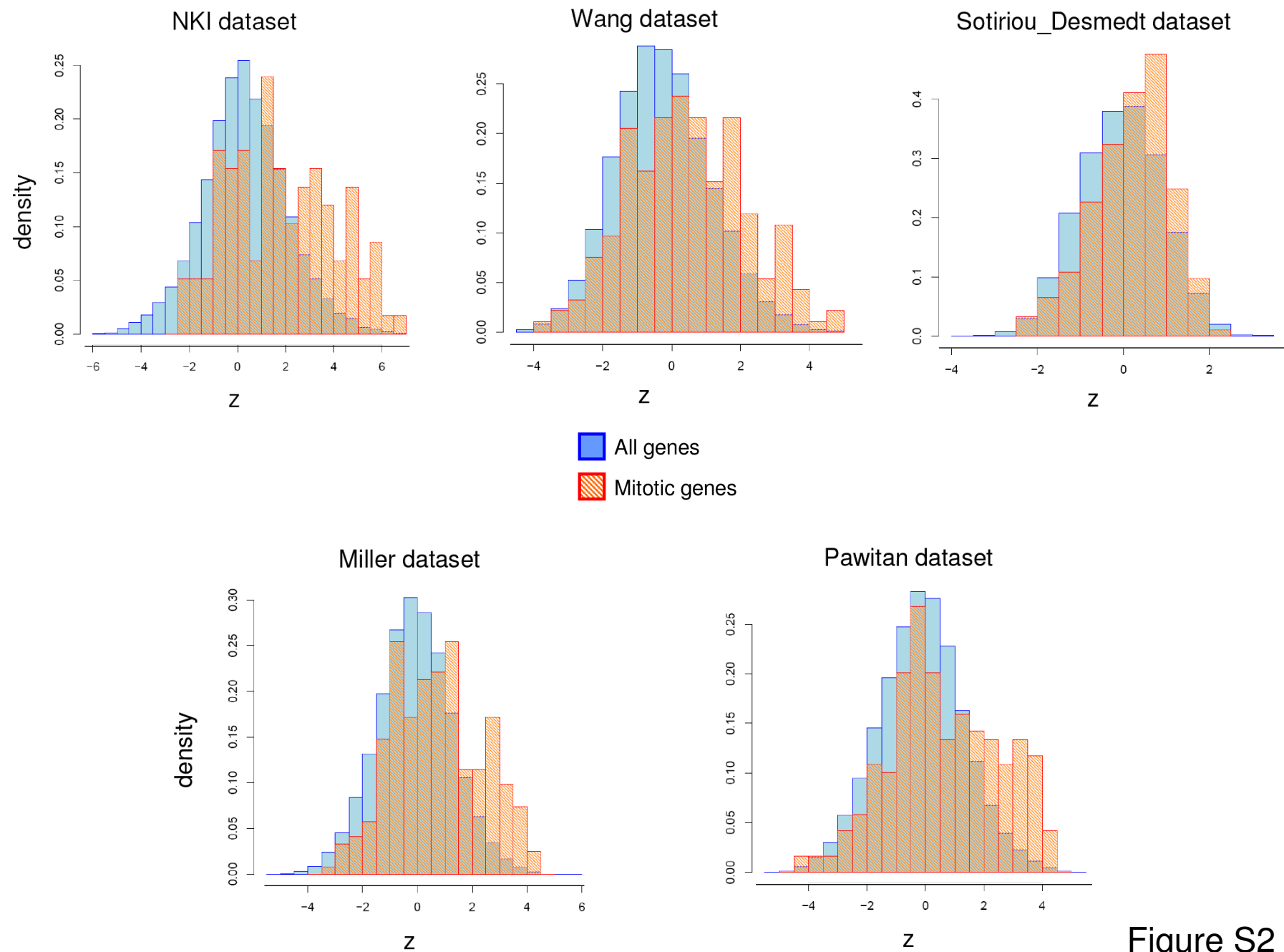

Figure S2

Supplement: Figure S2 — Distribution of the z-scores of the genes of the DM signature compared to the distribution of z-scores of all genes represented in five breast cancer datasets. Distribution of the z-scores of the genes of the DM signature compared to the distribution of z-scores of all genes represented in five breast cancer datasets. The z-scores were obtained using Cox univariate analysis. Note that the distribution of the signature genes is shifted towards positive values. (0.28 MB PDF) [file pone.0014737.s002.pdf]
